# One Year of Oral Immunotherapy Impacts the Gut Microbiota and Plasma Metabolome of Peanut‐Allergic Young Children

**DOI:** 10.1111/cea.14607

**Published:** 2024-11-27

**Authors:** Isabella Badolati, Ymke de Jong, Carina Uhl, Josefin Ullberg, Marleen Joustra, Ulrika Lorentzon Fagerberg, Caroline Nilsson, Anna Asarnoj, Eva Sverremark‐Ekström

**Affiliations:** ^1^ Department of Molecular Biosciences, The Wenner‐Gren Institute Stockholm University Stockholm Sweden; ^2^ Department of Clinical Science and Education, Södersjukhuset Karolinska Institutet Stockholm Sweden; ^3^ Sachs' Children and Youth Hospital Stockholm Sweden; ^4^ Department of Pediatrics Västmanland Hospital Västerås Sweden; ^5^ Centre for Innovation, Research and Education, Region Västmanland Västmanland Hospital Västerås Sweden; ^6^ Department of Women's and Children's Health Karolinska Institutet Stockholm Sweden; ^7^ Pediatric Allergy and Pulmonology Unit at Astrid Lindgren Children's Hospital Karolinska University Hospital Stockholm Sweden

**Summary**

- One‐year peanut OIT in young children seems to increase gut microbiota richness and Clostridia abundance.
- Plasma acylcarnitines and fatty acids follow opposite trajectories over time in OIT‐treated and untreated children.

To the Editor,

Oral immunotherapy (OIT) has become a promising treatment for peanut allergy to induce desensitisation or, in the best‐case, tolerance. Intervention in young children appears particularly favorable [1], possibly due to higher plasticity of their immune system. Gut microbes and their metabolites are known to play a role in food allergy [2, 3], but if and how they are involved in OIT is only beginning to be explored.

In this study, we aimed to evaluate the effects of 1‐year peanut OIT on the gut microbiota and metabolic profile of young children (aged 1–3 years) participating in the SmaChO trial [4] (Figure 1A). The first 34 children (out of 75) reaching 1‐year follow‐up were included, with 17 receiving OIT (OIT group) and 17 avoiding peanut (No OIT group). Faeces and peripheral blood were collected at baseline and 1‐year time‐points, along with demographic and clinical information. Informed, written consent was obtained from the participants' parents. All clinical, microbiota and metabolites' results mentioned but not shown in Figure 1 can be found in the online repository, at https://doi.org/10.5281/zenodo.14140191, together with methodological details.

**FIGURE 1:**
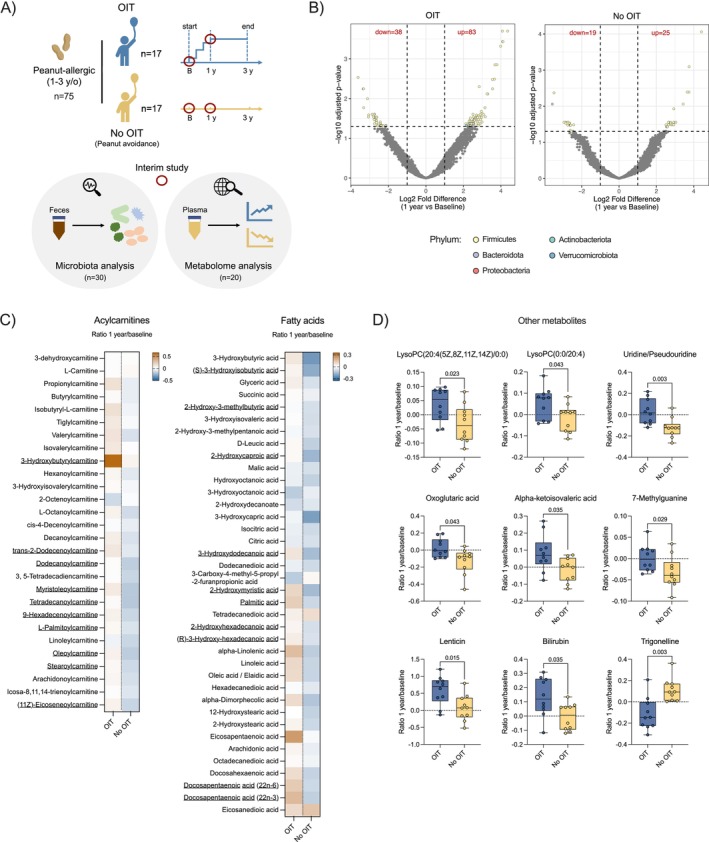
One year of OIT affects the gut microbiota and plasma metabolic profile of peanut‐allergic young children differently than continued peanut avoidance. (A) Scheme of the interim experimental study conducted at 1‐year follow‐up on 34 peanut‐allergic children (aged 1–3 years) from the SmaChO trial. Of these children, 17 received oral immunotherapy (OIT) and 17 avoided peanut (No OIT). Both gut microbiota (*n* = 30) and plasma metabolome (*n* = 20) were investigated at baseline and 1 year time‐points. (B) Volcano plots showing the differentially abundant amplicon sequence variants (ASVs) between 1 year and baseline, within the OIT and No OIT groups. Each dot denotes one ASV, with different colours depending on the phylum. DESeq2 analysis was performed to identify ASVs with significantly different abundance between time‐points. Up = log2 fold change > 1 and adjusted *p* < 0.05, down = log2 fold change < −1 and adjusted *p* < 0.05. (C) Heat maps illustrating the ratio between 1 year and baseline levels of 28 acylcarnitines (left) and 38 fatty acids (right) found in the OIT and No OIT children. Median values were log‐transformed to generate the heat maps, and a colorimetric scale was used for presentation purposes. To compare the two groups, the Mann–Whitney test was used, followed by multiple comparisons correction (Benjamini, Krieger and Yekutieli method). A false discovery rate (FDR) cut‐off of 0.05 was set but since the threshold was not passed by any of the metabolites, potentially due to small sample size, an unadjusted *p* < 0.05 was used to highlight individual metabolites (underlined in heat maps). (D) Box plots showing the ratios between 1 year and baseline levels of lysoPC(20:4(5Z,8Z,11Z,14Z)/0:0), lysoPC(0:0/20:4) and uridine/pseudouridine (top row), oxoglutaric acid, alpha‐ketoisovaleric acid and 7‐methylguanine (middle row), as well as lenticin, bilirubin and trigonelline (bottom row), in OIT and No OIT children. Values were log‐transformed prior to representation. Median and IQR are presented, with whiskers covering min‐to‐max values. Individual metabolites were selected based on the results of the Mann–Whitney test followed by multiple comparisons correction. A *p* < 0.05 was considered to filter, with the specific *p*‐values displayed in each plot. Adjusted *p*‐values for all metabolites can be found in the online repository, at https://doi.org/10.5281/zenodo.14140191. In (B), *n* = 15 for each group. In (C, D), *n* = 10 for each group.

Notably, after 1 year, the dose of peanut tolerated by OIT‐treated children was significantly higher compared to untreated children (5000 mg vs. 27.7 mg), indicating a high degree of desensitisation.

To study the gut microbiota, faeces from children with paired baseline and 1‐year samples available (15 from each group) were analysed, using 16S rRNA sequencing. At 1 year, the α‐diversity tended to be higher in the OIT group, where bacterial richness, in particular, increased significantly from baseline, consistent with previous findings in adults [5]. The β‐diversity revealed no differences in overall microbial composition between the two groups, which may be explained by the large interindividual variations typical of gut microbiota.

Over 500 amplicon sequence variants significantly differed in abundance between the groups at the 1‐year mark, with the majority being Firmicutes. Numerous genera within the Clostridia class, for example, *Faecalibacterium*, were significantly more abundant in the OIT group. An enrichment in Clostridia was previously shown not only in healthy compared to allergic individuals [6], but also following OIT [5], suggesting a link to the process of peanut desensitisation. Interestingly, when examining the change over time (1 year vs. baseline), the OIT group showed alterations in a substantial number of bacteria, while a considerably smaller change was observed in the No OIT group (Figure 1B), suggesting a slower maturation of their gut microbiota.

The plasma metabolome was investigated by liquid chromatography–mass spectrometry in 10 OIT and 10 No OIT children, randomly selected from the 17 in each group. A total of 217 known metabolites were identified. At 1‐year follow‐up, the metabolic profile differed between the groups; treated individuals exhibited a dispersed distribution in the principal component analysis plot, while untreated individuals formed a distinct, non‐overlapping cluster.

Both acylcarnitines and fatty acids displayed different trajectories over time based on whether or not the children received OIT (Figure 1C). In the OIT group, the overall trend was an increase from baseline to 1 year in these metabolites, while levels declined in the No OIT group. Albeit no difference reached statistical significance after multiple comparisons adjustment, several acylcarnitines and fatty acids had a strongly higher 1 year/baseline ratio in OIT children (*p* < 0.05, highlighted in the heat maps). Lower levels of these metabolites were previously associated with food allergy [7, 8], but our data adds to the existing knowledge, by looking at the temporal changes associated with OIT or the continuous development of peanut allergy. While fatty acids are known to directly influence immune responses, how acylcarnitines modulate immunity in the context of allergies remains unclear.

Another class of lipids, lysophosphatidylcholines (lysoPCs), exhibited distinct trajectories from baseline to 1‐year follow‐up in the two groups, and only increased upon OIT (Figure 1D). Prior studies found dysregulation of lysoPCs in food allergies, with higher levels in resolving compared to persistent disease [7]. Additionally, we noted a higher 1 year/baseline ratio in OIT‐treated children for metabolites like uridine/pseudouridine and bilirubin, whereas trigonelline showed the opposite pattern (Figure 1D). The pronounced increase in bilirubin upon OIT was particularly intriguing, as it may relate to the gut microbiota changes, due to the gut–liver axis. Also, bilirubin was previously linked to protection against allergic inflammation because of its capacity to negatively regulate ILC2s [9].

Plasma levels of short‐chain fatty acids (SCFAs), including acetate, propionate, butyrate and isobutyrate, along with two of their precursors, showed no major differences between OIT and No OIT groups. This was in contrast to earlier research attributing tolerogenic properties to SCFAs and showing variations between allergic and non‐allergic individuals [3]. However, we measured these metabolites in a relatively small sample and in plasma, not faeces, where results could differ.

While this work has limitations, such as including only 34 out of 75 children from the SmaChO study, and further subsampling for each experimental technique, it also has key strengths. We used a unique, well‐characterised cohort, in which both OIT‐treated and untreated groups were analysed for microbiota and metabolome over time, which was not done in previous reports. Although one might speculate that peanut consumption during OIT directly influenced gut microbes and plasma metabolites, this is unlikely, as even the highest dose (285 mg) represents a minimal fraction of daily dietary intake.

In conclusion, we show that OIT in young children, after 1 year, induces gut microbiota as well as plasma metabolic changes, which are not observed in their untreated counterpart and may be linked to peanut desensitisation. These findings expand the so‐far limited knowledge of the mechanisms underlying peanut OIT.

## Author Contributions

I.B., E.S.E., A.A., C.N. and U.L.F. conceptualised the study. I.B. and M.J. performed laboratory work, and C.U. and J.U. provided all clinical data. I.B. and Y.d.J. performed data analysis. I.B. and E.S.‐E. wrote the manuscript, and all authors contributed with comments and revision.

## Conflicts of Interest

E.S.‐E has received honoraria for lectures and a research grant for another project from BioGaia AB. C.N. has received lecture fees from MEDA, GSK, ThermoFisher and ALK, grants to institution from Aimmune Therapeutics, a Nestlé Health Science company, and material for IgE analyses from ThermoFisher. A.A. has received lecture fees from Orion Pharma, Nestlé, Semper, ThermoFisher and ALK, and advisory board fees from Novartis, Sanofi, Danone and Aimmune Therapeutics. C.U. received lecture fees from Aimmune Therapeutics. U.L.F. received lecture fees from Nestlé. No conflict of interest reported from the other authors.

## Data Availability

Supporting data are publicly available in the online repository, at https://doi.org/10.5281/zenodo.14140191. The 16S rRNA data have been deposited in the NCBI Sequence Read Archive (BioProject ID: PRJNA1125362). All other raw data are available upon request.
